# Effet des sanctions économiques sur la qualité de la prise en charge hospitalière de la malnutrition aiguë sévère au Niger: une étude rétrospective multicentrique

**DOI:** 10.11604/pamj.2026.53.131.49730

**Published:** 2026-03-17

**Authors:** Maimouna Halidou Doudou, Sakina Habou, Aichatou Achirou

**Affiliations:** 1 Université Privée Africaine pour le Développement, Niamey, Niger,; 2 Direction de la Recherche du Ministère de la Santé et de l'Hygiène Publique, Niamey, Niger

**Keywords:** Sanctions économiques, malnutrition aiguë sévère, qualité des soins, disparités régionales, Niger, résilience du système de santé, Economic sanctions, severe acute malnutrition, quality of care, regional disparities, Niger, health system resilience

## Abstract

**Introduction:**

en 2023, le Niger a été soumis à des sanctions économiques internationales à la suite d'une instabilité politique, entraînant des perturbations majeures dans les chaînes d'approvisionnement et la continuité des services de santé. Cette étude visait à évaluer l'effet de ces sanctions sur la qualité de la prise en charge hospitalière de la malnutrition aiguë sévère (MAS) dans les centres thérapeutiques du pays.

**Méthodes:**

il s'agit d'une étude rétrospective multicentrique réalisée de janvier 2022 à décembre 2023 dans sept centres de récupération nutritionnelle intensive (CRENI) répartis dans six régions du Niger (Dosso, Maradi, Niamey, Tahoua, Tillabéry et Zinder). Les données ont été extraites des registres hospitaliers à l'aide d'un outil standardisé. Les indicateurs de qualité des soins (taux d'admission, de guérison, de décès, durée moyenne de séjour et taux d'abandon) ont été comparés avant et après l'imposition des sanctions. Des entretiens qualitatifs auprès du personnel ont permis d'explorer les difficultés opérationnelles et les ruptures d'approvisionnement.

**Résultats:**

au total, 6354 cas de MAS ont été admis sur la période d'étude. En comparaison avec 2022, l'année 2023 a enregistré une augmentation de 22% des admissions, une baisse du taux de guérison (de 83,4% à 74,6%) et une hausse de la mortalité (de 9,1% à 14,3%). Des disparités régionales marquées ont été observées: les CRENI urbains (Niamey, Maradi) ont mieux résisté aux perturbations que ceux des zones périphériques (Tahoua, Tillabéry, Zinder), plus affectés par les ruptures et le manque de personnel.

**Conclusion:**

les sanctions économiques ont significativement perturbé le système de prise en charge nutritionnelle, compromettant la qualité des soins et accentuant les inégalités régionales. Le renforcement de la résilience logistique et la protection des services essentiels s'avèrent indispensables pour préserver la survie infantile en contexte de crise.

## Abstract (en)

**Introduction:**

in 2023, Niger was subjected to international economic sanctions following political instability, resulting in major disruptions to supply chains and continuity of health services. This study aimed to assess the effect of these sanctions on the quality of hospital management of severe acute malnutrition (SAM) in therapeutic centres across the country.

**Methods:**

this multicenter retrospective study was conducted from January 2022 to December 2023 in seven intensive nutritional rehabilitation centres (CRENI) located in six regions of Niger (Dosso, Maradi, Niamey, Tahoua, Tillabéry, and Zinder). Data was extracted from hospital registers using a standardised tool. Quality-of-care indicators (admission rate, recovery rate, mortality rate, average length of stay, and default rate) were compared before and after the imposition of sanctions. Qualitative interviews with health staff were also conducted to explore operational challenges and supply chain disruptions.

**Results:**

a total of 6,354 SAM cases were admitted during the study period. Compared with 2022, the year 2023 recorded a 22% increase in admissions, a decrease in the recovery rate (from 83.4% to 74.6%), and an increase in mortality (from 9.1% to 14.3%). Marked regional disparities were observed: urban CRENIs (Niamey, Maradi) were more resilient to disruptions than those in peripheral areas (Tahoua, Tillabery, Zinder), which were more severely affected by stockouts and staff shortages.

**Conclusion:**

economic sanctions significantly disrupted the nutritional care system, undermining the quality of services and exacerbating regional inequalities. Strengthening logistical resilience and safeguarding essential health services are critical to preserving child survival in crisis contexts.

## Introduction

Le monde contemporain connaît une instabilité politique croissante, marquée par la recrudescence des coups d'État, des tensions géopolitiques et des conflits régionaux. En réaction à ces situations, les organisations internationales et régionales recourent fréquemment aux sanctions économiques comme instrument diplomatique destiné à rétablir l'ordre constitutionnel, dissuader les violations des droits humains et promouvoir la bonne gouvernance [1-3]. Toutefois, si ces mesures visent des objectifs politiques légitimes, elles peuvent engendrer des effets délétères sur les systèmes de santé, en limitant l'accès aux ressources financières, logistiques et pharmaceutiques nécessaires au maintien des services essentiels [4-6].

Pour comprendre l'impact des sanctions sur la qualité des soins, le modèle de Donabedian [7] constitue un cadre analytique pertinent: il distingue trois composantes interdépendantes: i) la structure (ressources humaines, infrastructures, approvisionnement); ii) les processus (organisation et prestation des soins); iii) les résultats (guérison, mortalité, satisfaction). Les sanctions économiques agissent principalement sur la structure du système de santé, en restreignant les importations, les financements et les partenariats techniques, ce qui perturbe ensuite les processus de soins et se traduit par une dégradation des résultats cliniques. Ce cadre théorique permet donc d'articuler l'analyse des effets indirects des sanctions sur la prise en charge hospitalière de la malnutrition au Niger [8-10].

Plusieurs travaux ont documenté les conséquences des sanctions économiques sur la santé publique. En Iran, les restrictions financières ont entraîné des pénuries de médicaments essentiels et une hausse des décès liés aux maladies chroniques [11-13]. En Syrie, les sanctions ont compromis les chaînes d'approvisionnement humanitaire, aggravant la morbidité infantile et la malnutrition [14,15]. Au Venezuela, la contraction économique induite par les sanctions américaines a conduit à une augmentation de la mortalité infantile et à une insécurité alimentaire généralisée [16]. Des études de l'Organisation mondiale de la Santé (OMS) et de Human Rights Watch ont également mis en évidence un effet systémique des sanctions sur la résilience des systèmes de santé, notamment par la réduction du financement extérieur, la fuite du personnel qualifié et la détérioration de la qualité des soins [3,17]. Malgré cette littérature croissante, peu de recherches empiriques ont examiné les effets directs des sanctions sur la qualité de la prise en charge hospitalière en Afrique de l'Ouest, et aucune, à notre connaissance, n'a porté spécifiquement sur la malnutrition aiguë sévère. Cette lacune justifie l'intérêt scientifique d'une telle étude. Au Niger, la malnutrition infantile demeure une urgence de santé publique. Selon les enquêtes démographiques et de santé successives, la prévalence de la malnutrition aiguë globale (MAG) reste supérieure aux seuils critiques de l'OMS, dépassant souvent 10 % chez les enfants de moins de cinq ans [18-20]. Malgré l'adoption de la Politique nationale de sécurité nutritionnelle (2017-2025) [21] et la mise en œuvre du Protocole national de prise en charge de la MAS [22], la couverture géographique et la disponibilité des intrants nutritionnels demeurent limitées.

Le coup d'Etat militaire du 26 juillet 2023 a entraîné une série de sanctions économiques et financières imposées par la Communauté économique des États de l'Afrique de l'Ouest (CEDEAO) et l'Union économique et monétaire ouest-africaine (UEMOA), incluant la fermeture des frontières, la suspension des transactions bancaires et le gel des avoirs publics. Ces mesures ont restreint les importations de produits pharmaceutiques, les flux logistiques et les financements des partenaires techniques et financiers, risquant ainsi de compromettre la continuité des soins hospitaliers, en particulier dans les CRENI. Sur la base du modèle de Donabedian [7], nous postulons que les sanctions économiques post-juillet 2023 ont fragilisé la structure (ruptures d'intrants, manque de personnel), perturbé les processus (retards de prise en charge, surcharge des équipes) et altéré les résultats (baisse des taux de guérison, hausse de la mortalité). L'objectif de la présente étude est donc de déterminer si les sanctions économiques imposées au Niger ont eu un impact mesurable sur la qualité de la prise en charge de la malnutrition aiguë sévère dans les structures hospitalières, à travers l'analyse comparative d'indicateurs de performance avant et après les sanctions.

## Méthodes

**Type et lieu de l'étude:** il s'agit d'une étude observationnelle rétrospective multicentrique, réalisée entre janvier 2022 et décembre 2023 dans sept (7) CRENI répartis dans six régions du Niger: Dosso, Maradi, Niamey, Tahoua, Tillabéry et Zinder. Les régions de Diffa et d'Agadez ont été exclues en raison de l'insécurité persistante qui rendait la collecte des données non réalisable. Cette approche multicentrique a été retenue afin d'assurer la représentativité géographique et structurelle du système de prise en charge hospitalière de la MAS et d'accroître la validité externe des résultats. L'étude s'inscrit dans le cadre conceptuel de Donabedian sur la qualité des soins, considérant trois dimensions: i) la disponibilité des ressources humaines, intrants thérapeutiques et équipements; ii) l'organisation et continuité de la prise en charge; et iii) les indicateurs de performance clinique (guérison, mortalité). Outre l'exploitation des registres et rapports statistiques des CRENI, une composante qualitative a été intégrée à la méthodologie afin d'enrichir l'interprétation des résultats quantitatifs.

**Population d'étude et critères d'inclusion:** l'étude a porté sur l'ensemble des enfants âgés de moins de cinq ans admis pour MAS dans les CRENI sélectionnés au cours des trois périodes suivantes: i) semestre 2, 2022 (période de référence avant sanctions); ii) semestre 1, 2023 (période immédiatement avant sanctions); iii) semestre 2, 2023 (période post-sanctions, marquée par les mesures économiques de la CEDEAO et de l'UEMOA). Les critères d'inclusion étaient: i) enfants âgés de 0 à 59 mois répondant aux critères d'admission pour MAS selon le Protocole national de prise en charge de la malnutrition aiguë sévère (2016) [15]; ii) dossiers médicaux complets contenant les informations nécessaires aux indicateurs retenus. Les dossiers présentant des données manquantes sur les issues thérapeutiques (guérison, transfert, décès) ont été exclus de l'analyse. Pour la partie qualitative, les données ont été recueillies auprès de douze (12) prestataires, par voie électronique (guide d'entretien via courriel et téléphone), puis anonymisées et codées thématiquement selon les grands axes du cadre conceptuel de Donabedian [7].

**Sources et collecte des données:** la collecte a été effectuée à partir des registres d'admission et de suivi des CRENI, des rapports mensuels d'activités des directions régionales de la santé publique, des fiches de gestion des intrants nutritionnels et thérapeutiques, et, lorsque disponible, des rapports de supervision et d'inventaire pharmaceutique. Un formulaire standardisé d'extraction a été élaboré et prétesté. Deux enquêteurs formés ont travaillé par région, sous la supervision d'un pédiatre référent régional. Un double contrôle de qualité et de cohérence des données a été réalisé, d'abord sur le terrain, puis au niveau central à Niamey. Un guide d'entretien semi-structuré a été élaboré et adressé aux prestataires de soins et responsables de service des CRENI.

### Variables étudiées

**Variables principales (indicateurs de résultat):** le taux de guérison/de sortie réussie (% des enfants guéris ou transférés avec amélioration) a été calculé selon la formule suivante [4]: Taux de guéris (%) = (nombre total de guéris/nombre total de sorties) x 100. Selon le protocole national de prise en charge de la malnutrition aiguë [15], cet indicateur permet d'apprécier la qualité des soins et l'adhésion/collaboration de l'accompagnante/mère de l'enfant malnutri. Le taux de mortalité/létalité (% des enfants décédés pendant la prise en charge) est calculé selon la formule suivante [4]: Létalité (%) = (nombre total de décès/nombre total de sorties) x 100. Cet indicateur permet d'apprécier la qualité des soins et le recours tardif aux soins de santé. Ces deux indicateurs ont été calculés selon les formules du Protocole national de prise en charge de la MAS [22] et comparés aux seuils de performance du SPHERE.

**Variables structurelles et de processus:** i) disponibilité des intrants thérapeutiques: durée et fréquence de rupture de F75, F100, Plumpy'Nut, Resomal, médicaments essentiels; ii) disponibilité du matériel anthropométrique: balance, toise, MUAC; ressources humaines: nombre et catégorie de personnel (médecins, infirmiers, nutritionnistes, stagiaires) par centre; iii) charge de travail: ratio enfants admis/agent de santé.

**Les données qualitatives:** l'entretien à travers un guide d'entretien semi-structuré portait principalement sur les ressources humaines disponibles et les conditions de travail, les difficultés rencontrées dans la prise en charge pendant la période de sanctions, les ruptures d'intrants thérapeutiques et logistiques et les stratégies d'adaptation mises en œuvre par les équipes pour maintenir la qualité des soins.

**Plan d'analyse des données:** les données ont été saisies et analysées à l'aide du logiciel STATA version 17.0. L'analyse descriptive a été faite à l'aide des moyennes, écarts-types, proportions et intervalles de confiance à 95%. L'analyse comparative par les comparaisons entre périodes avant et après sanctions à l'aide du test t de Student pour échantillons indépendants et les comparaisons ajustées sur la saisonnalité: semestre équivalent 2022 vs. 2023. Le seuil de significativité considéré est < 0,05. Sur le plan qualitatif, l'analyse de contenu thématique a été conduite manuellement pour identifier les récurrences, convergences et divergences dans les perceptions des prestataires.

**Considérations éthiques:** l'étude a reçu l'approbation du Comité National d'Éthique pour la Recherche en Santé du Niger et une autorisation a été obtenue auprès du Ministère de la Santé et de L'hygiène Publique, ainsi que des directeurs régionaux de la santé publique et des directeurs d'hôpitaux concernés. Les données ont été anonymisées avant l'analyse, et aucun identifiant individuel n'a été conservé.

**Limites et biais potentiels de l'étude:** cette étude présente certaines limites inhérentes à son caractère rétrospectif. Les données proviennent des registres hospitaliers de sept CRENI, ce qui peut introduire un biais de sélection, les enfants non admis n'étant pas représentés. La qualité variable des enregistrements et les ruptures de matériel peuvent également générer un biais d'information.

Par ailleurs, des facteurs contextuels non mesurés (autres crises sanitaires, différences régionales de ressources) peuvent avoir influencé les indicateurs observés. Enfin, l'absence d'analyse multivariée limite le contrôle des facteurs de confusion. Ces éléments invitent à interpréter les résultats avec prudence, tout en soulignant la cohérence globale des tendances observées.

## Résultats

**Figure 1:**
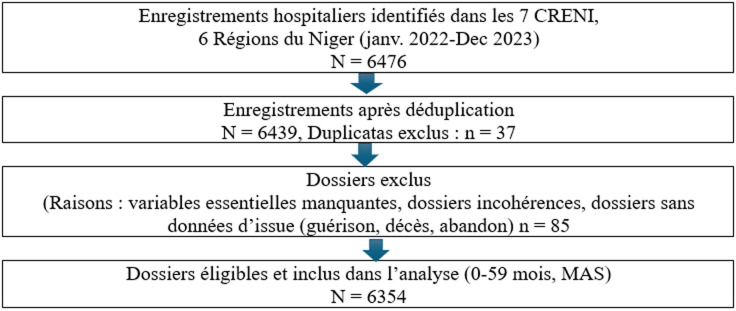
diagramme de flux des dossiers hospitaliers inclus dans l'étude rétrospective multicentrique sur la malnutrition aiguë sévère au Niger, 2022-2023

**Diagramme de flux des dossiers hospitaliers:** le flux des enfants inclus dans l'étude est résumé dans la Figure 1. Au total, 6 476 enregistrements provenant des registres hospitaliers des sept CRENI répartis dans six régions du Niger (Dosso, Maradi, Niamey, Tahoua, Tillabéry et Zinder) ont été initialement identifiés pour la période de janvier 2022 à décembre 2023. Après élimination de 37 doublons, le nombre d'enregistrements valides s'est établi à 6 439 dossiers. Parmi ceux-ci, 85 dossiers ont été exclus en raison de variables essentielles manquantes, de données incohérentes ou de l'absence d'information sur l'issue du traitement (guérison, décès ou abandon). Ainsi, 6 354 dossiers complets et éligibles d'enfants âgés de 0 à 59 mois atteints de MAS ont été inclus dans l'analyse principale.

**Caractéristiques générales des structures étudiées:** au total, sept (7) CRENI répartis dans six (6) régions du Niger ont été inclus: Dosso, Maradi, Niamey, Tahoua, Tillabéry et Zinder. Les CRENI différaient sensiblement en capacité d'accueil et en ressources humaines, mais tous appliquaient le protocole national de prise en charge de MAS. Douze (12) prestataires de soins et responsables de service des CRENI ont été inclus.

**Évolution des admissions de cas de malnutrition:** la comparaison entre les périodes avant et après sanctions (semestre 1 et semestre 2, 2023) montre une augmentation significative du nombre moyen d'admissions dans plusieurs structures (Tableau 1). Les hausses les plus marquées ont été observées: i) à l'hôpital national de Zinder (+219,3 admissions; p = 0,0019); ii) à l'hôpital national de Niamey (HNN) (+42,5 admissions; p = 0,0022); iii) au centre hospitalier régional (CHR) de Tahoua (+45,7 admissions; p = 0,0002). En revanche, les CHR de Maradi et de Tillabéry n'ont pas montré de variation significative (p > 0,05).

**Tableau 1:** comparaison des moyennes de nouvelles admissions avant et après les sanctions pour le premier et le second semestre 2023 au Niger

Structures sanitaires	Semestre 1, 2023 (sans sanction)	Semestre 2, 2023 (avec sanction)		
	Moyenne (DS)	Moyenne (DS)	Différence moyenne	P
	**Nombre d'admission dans les CRENI**			
CHR poudrière	89,3 (25,6)	121,5 (34,9)	+32,2	**0,0494**
CHR Dosso	38,2 (16,1)	51,2 (10,2)	+13,0	0,0677
CHR Tahoua	58,0 (18,2)	103,7 (9,7)	+45,7	**0,0002**
Hôpital national Zinder	234,0 (59,7)	453,3 (130,8)	+219,3	**0,0019**
Hôpital national Niamey	80,5 (23,8)	123,0 (15,6)	+42,5	**0,0022**
CHR Maradi	125,2 (50,0)	143,0 (26,0)	+17,8	0,2317
CHR Tillabéry	53,7(21,1)	65,2 (21,7)	+11,5	0,1869

Lorsque la comparaison intègre la saisonnalité (semestre 2, 2022 vs semestre 2, 2023), la tendance globale d'augmentation se maintient, bien que non significative pour la majorité des centres (Tableau 2). Une exception notable est observée au CHR de Maradi, où les admissions ont diminué significativement en 2023 (p = 0,0433). Sur le plan qualitatif, les entretiens informels menés avec les prestataires des CRENI ont révélé une préoccupation commune quant à la hausse du flux d'admissions et la pression accrue sur les équipes, sans renforcement concomitant du personnel depuis les sanctions économiques à la suite du coup d'Etat de juillet 2023.

**Tableau 2:** comparaison des moyennes de nouvelles admissions avant et après les sanctions pour les seconds semestres 2022 et 2023 au Niger

Structures sanitaires	Semestre 2, 2022 (sans sanction)	Semestre 2, 2023 (avec sanction)		
	Moyenne (DS)	Moyenne (DS)	Différence moyenne	P
	**Nombre d'admission dans les CRENI**			
CHR poudrière	94,7 (37,5)	121,5 (34,9)	+26,8	0,1141
CHR Dosso	48,0 (22,5)	51,2 (10,2)	+3,2	0,3819
CHR Tahoua	87,3 (41,4)	103,7 (9,7)	+16,4	0,1846
Hôpital national Zinder	665,2 (306,4)	453,3 (130,8)	-211,9	0,0905
Hôpital national Niamey	103,8 (35,5)	123 (15,6)	+19,2	0,1272
CHR Maradi	215,2 (88,7)	143,0 (26,0)	-72,2	**0,0433**
CHR Tillabéry	62,8 (24,9)	65,2 (21,7)	+2,4	0,4330
Centre hospitalier régional; différence de moyenne = moyenne second semestre				

**Indicateurs de performance clinique:** concernant le taux de sortie avec succès (guérison ou transfert vers une structure sanitaire de premier niveau), les résultats dans le Tableau 3 indiquent que les meilleures performances ont été enregistrées au cours du premier semestre 2023, à l'exception de l'HNN et du CHR de Maradi, où les proportions étaient légèrement inférieures. Toutefois, les différences observées entre les deux semestres n'étaient pas statistiquement significatives dans l'ensemble des structures (p > 0,05). En revanche, une hausse du taux de mortalité chez les enfants malnutris sévères a été observée dans cinq (5) des sept (7) structures sanitaires au cours du deuxième semestre 2023 (période sous sanctions). Cette augmentation était statistiquement significative (p < 0,05) dans les CHRs de Tahoua, Maradi, Tillabéry ainsi qu'à l'HNN. À l'inverse, une mortalité plus faible a été enregistrée au cours de la même période au CHR de Dosso et à l'hôpital national de Zinder (HNZ), sans différence significative.

**Tableau 3:** indicateurs de performance dans les centres de récupération nutritionnelle intensive avant et après les sanctions pour le premier et le second semestre 2023 au Niger

Structures sanitaires	Semestre 1, 2023 (sans sanction)	Semestre 2, 2023 (avec sanction)	-	-
	Moyenne (DS)	Moyenne (DS)	Différence	P
**Proportion des enfants sortis guéris ou transférés**				
CHR poudrière	91,7 (25,6)	75,5 (31,5)	-16,2	0,1201
CHR Dosso	91,1 (2,9)	88,3 (3,5)	-2,8	0,0788
CHR Tahoua	92,2 (4,6)	91,4 (3,1)	-0,8	0,3591
Hôpital national Zinder	96,3 (1,2)	96,5 (1,7)	+0,2	0,4035
Hôpital national Niamey	91,0 (4,5)	90,1 (2,0)	-0,9	0,3233
CHR Maradi	91,5 (5,8)	92,8 (2,5)	+1,3	0,3107
CHR Tillabéry	97,6 (3,0)	95,7 (2,3)	-1,9	0,1331
**Proportion des enfants décédés**				
CHR poudrière	7,2 (3,5)	8,3 (1,3)	+1,1	0,2636
CHR Dosso	5,5 (2,5)	4,6 (1,6)	-0,9	0,2542
CHR Tahoua	4,5 (2,3)	7,7(3,6)	+3,2	0,0556
Hôpital national Zinder	3,1 (0,9)	2,6 (1,1)	-0,5	0,2157
Hôpital national Niamey	4,6 (2,9)	7,1(1,3)	+2,5	0,0485
CHR Maradi	3,2 (1,3)	5,0 (1,3)	+1,8	0,022
CHR Tillabéry	1,7(1,6)	3,8 (2,3)	+2,1	0,0502

L'analyse du taux de guérison dans les CRENI (Tableau 4) a révélé une meilleure performance au cours du deuxième semestre 2022, période précédant les sanctions économiques, dans la majorité des structures sanitaires. Deux exceptions ont été observées: l'hôpital national de Zinder (HNZ) et le CHR de Tahoua, où les taux de guérison étaient supérieurs en 2023. Toutefois, une différence statistiquement significative n'a été constatée que dans le CHR de Maradi (p = 0,002), indiquant une baisse marquée du taux de guérison dans cette structure durant la période sous sanctions. En ce qui concerne le taux de mortalité, les résultats ont montré qu'il était plus élevé dans cinq (5) des sept (7) structures au deuxième semestre 2022, bien que les écarts observés ne soient pas statistiquement significatifs. Cependant, une mortalité significativement plus faible a été notée au CHR de Maradi durant cette même période (p = 0,005), tandis qu'à l'HNN, la baisse observée en 2022 comparée à 2023 était non significative (p = 0,316).

**Tableau 4:** indicateurs de performance dans les centres de récupération nutritionnelle intensive avant et après les sanctions pour les seconds semestres 2022 et 2023 au Niger

Structures sanitaires	Semestre 2, 2022 (sans sanction)	Semestre 2, 2023 (avec sanction)		
	Moyenne (DS)	Moyenne (DS)	Différence	P
**Proportion des enfants sortis guéris ou transférés**				
CHR Poudrière	90,6 (4,6)	75,5 (31,5)	-15,1	0,1353
CHR Dosso	90,4 (5,4)	88,3 (3,5)	-2,1	0,2197
CHR Tahoua	88,8 (4,1)	91,4 (3,1)	+2,6	0,1223
Hôpital national Zinder	95,3 (1,6)	96,5 (1,7)	+1,2	0,1133
Hôpital national Niamey	91,4 (3,8)	90,1(2,0)	-1,3	0,2317
CHR Maradi	96,6 (0,6)	92,8 (2,5)	-1,3	**0,0024**
CHR Tillabéry	96,6 (3,6)	95,7 (2,3)	-0,9	0,4146
**Proportion des enfants décédés**				
CHR poudrière	8,4 (2,6)	8,3 (1,3)	-0,1	0,4583
CHR Dosso	6,8 (4,2)	4,6 (1,6)	-2,2	0,1274
CHR Tahoua	7,8 (4,4)	7,7 (3,6)	-0,1	0,4798
Hôpital national Zinder	3,5 (0,6)	2,6 (1,1)	-0,9	0,0604
Hôpital national Niamey	6,3 (4,0)	7,1 (1,3)	+0,8	0,3159
CHR Maradi	3,2 (0,2)	5,0 (1,3)	+1,8	**0,0054**
CHR Tillabéry	3,6 (2,5)	3,8 (2,3)	-0,2	0,4370

Centre hospitalier régional; différence de moyenne = moyenne second semestre 2023-second semestre 2023; P-value du test de Student

**Disponibilité des intrants thérapeutiques et des médicaments:** des ruptures d'intrants essentiels ont été signalées dans l'ensemble des structures sanitaires évaluées, avec des durées et des fréquences variables selon les sites. La durée médiane était de 90 jours, variant entre 75 (CHR Poudrière) et 180 jours (CHR Tillabéry). Ces ruptures ont été observées à partir du mois d'octobre 2023 dans la majorité des CRENI, à l'exception des CHRs de Maradi et de Tillabéry, où elles ont été signalées plus tardivement. Les produits concernés incluent à la fois les médicaments essentiels et les intrants thérapeutiques spécifiques à la prise en charge de la malnutrition aiguë sévère, tels que présentés dans le Tableau 5. Les ruptures ont touché des médicaments comme les antibiotiques, les antipaludéens et l'acide folique; des intrants nutritionnels (F75, F100 et Plumpy'Nut). Les manques d'information sur la disponibilité des médicaments pour les années antérieures à la sanction économique n'ont pas permis de faire les comparaisons entre périodes.

**Tableau 5:** situation des ressources humaines dans les centres de récupération nutritionnelle intensive

Région / structure	Données collectées 2023		Annuaires statistiques de santé 2022 et 2023			
	Nombre d'agents CRENI	Ratio enfants/agent	Effectif CRENI 2022	Effectif CRENI 2023	Évolution (%)	Ratio enfants/agent
Hôpital national de Niamey	11	8	45	45	0 %	4–5
Hôpital national de Zinder	9	11	9	11	+22 %	11 (2022) → 9 (2023)
CHR Maradi	60	8	60	56	–6,7 %	8–9
CHR Tahoua	13	8	38	36	–5,3 %	7–8
CHR Tillabéry	19	3	20	22	+10 %	3–4
CHR Dosso	28	6	24	25	+4,1 %	5–6
CHR poudrière (Niamey)	12	4	18	19	+5,6 %	4–5


Annuaires statistiques 2022 et 2023 [23,24] Agents de santé toutes catégories confondues; Centre hospitalier régional; Approximative

**Disponibilité des outils anthropométriques:** tous les CRENI dans les six (6) régions disposaient de matériel (balances, toises, MUAC) en quantité suffisante pour le dépistage de la malnutrition en fin d'année 2023. Aucune rupture et aucun équipement défectueux n'ont été signalé.

**Ressources humaines disponibles:** dans le Tableau 5, la triangulation des données issues de l'enquête 2023 et des annuaires statistiques de santé 2022-2023 [23,24] a mis en évidence une tendance convergente de stagnation, voire de baisse des effectifs dans les CRENI des régions les plus sollicitées, notamment Maradi, Tahoua et Zinder. À Maradi, l'effectif est passé de 60 à 56 agents (-6,7%) pour une activité mensuelle moyenne de 451 cas, soit un ratio de 8 enfants par agent. À Tahoua, les effectifs ont reculé de 38 à 36 agents (-5,3%) avec un ratio similaire (7-8), tandis qu'à Zinder, malgré une légère hausse du personnel (+22%, de 9 à 11 agents), la charge de travail demeure la plus élevée du pays (11 enfants par agent). À l'inverse, les structures de Tillabéry, Dosso et de Poudrière présentent une évolution plus favorable: les effectifs y étaient en légère hausse (+4 à +10%) et les ratios plus équilibrés (3 à 5 enfants par agent) traduisent une meilleure disponibilité du personnel.

**Difficultés majeures rapportées sur le terrain:** les difficultés recensées au niveau des CRENI pendant la période post-sanctions peuvent être regroupées en trois grandes catégories: i) logistique et approvisionnement: les ruptures fréquentes et prolongées des intrants nutritionnels (F75, F100, Plumpy'Nut, Resomal) et des médicaments essentiels (amoxicilline, quinine, antipaludéens injectables); l'allongement des délais de livraison depuis les partenaires (Fonds des Nations unies pour l'enfance (UNICEF), Programme alimentaire mondial (PAM), Médecins sans frontières (MSF)) et l'insuffisance de moyens de transport pour la distribution régionale; ii) ressources humaines et charge de travail : la surcharge de travail était citée plusieurs fois par les prestataires comme difficulté et entrainant une fatigue physique et émotionnelle. Ces contraintes structurelles ont entraîné des répercussions directes sur la qualité du processus de soins, notamment en rallongeant la durée moyenne de séjour et en réduisant la disponibilité des soins d'urgence.

**Changements observés dans les circuits d'approvisionnement:** avant les sanctions, les produits nutritionnels et pharmaceutiques destinés aux CRENI provenaient essentiellement de deux circuits: le canal institutionnel (direction des approvisionnements du MSHP) et le canal des partenaires humanitaires (UNICEF, PAM, OMS, MSF). Après les sanctions, une restructuration partielle des circuits d'approvisionnement a été observée: i) centralisation accrue à Niamey: les commandes internationales étant bloquées, la majorité des stocks transitait par la capitale, provoquant des retards dans les livraisons régionales ; ii) recours accru aux stocks d'urgence des ONG et des programmes régionaux, notamment à Zinder, Maradi et Tahoua; ii) mise en place de circuits parallèles informels, parfois via des commandes locales ou transfrontalières non officielles (Nigeria, Tchad), pour pallier les ruptures prolongées; iii) diminution du soutien logistique international en raison des restrictions bancaires et de la suspension temporaire de plusieurs accords de financement. Ces changements ont entraîné une vulnérabilité accrue du système logistique, dépendant davantage d'interventions ponctuelles que d'un mécanisme d'approvisionnement structuré.

## Discussion

Plusieurs précautions ont été prises pour renforcer la validité des résultats: i) l'harmonisation des outils de collecte et supervision du terrain; ii) la vérification croisée des sources (registre, rapport, fiche de stock) et le contrôle des biais de saisonnalité et d'exclusion régionale (zones d'insécurité); iii) la formation préalable des agents de saisie afin d'uniformiser la définition des variables (critères de MAS, guérison, abandon, décès); iv) les critères d'inclusion et d'exclusion ont été appliqués de façon homogène sur tous les sites, et les données incomplètes ont été écartées pour éviter les biais de mesure; v) les bases de données consolidées ont ensuite été revues et validées par la direction des études et de la programmation du ministère de la santé publique, garantissant la cohérence et la comparabilité interrégionale des résultats.

Cette étude multicentrique constitue la première analyse empirique de l'impact des sanctions économiques de 2023 sur la qualité de la prise en charge hospitalière de la MAS au Niger. Les résultats révèlent une augmentation des admissions et une hausse de la mortalité hospitalière après l'imposition des sanctions, en lien avec les perturbations des chaînes d'approvisionnement en intrants thérapeutiques et la réduction des ressources humaines disponibles. Des disparités régionales marquées ont été observées, illustrant des inégalités de résilience liées à la disponibilité des ressources, à la gouvernance locale et au soutien des partenaires humanitaires.

Cette étude présente certaines limites méthodologiques qu'il convient de considérer dans l'interprétation des résultats. Le caractère rétrospectif de la collecte, fondée sur les registres hospitaliers, peut avoir introduit un biais d'information lié à la qualité variable des données. La période d'observation limitée (janvier 2022 - décembre 2023) restreint la mesure des effets à long terme des sanctions économiques. L'exclusion des régions d'Agadez et de Diffa, pour raisons sécuritaires, réduit la représentativité nationale et pourrait avoir sous-estimé l'impact global. Enfin, l'étude met en évidence une corrélation plutôt qu'une causalité directe entre sanctions et performance clinique, et la composante qualitative restreinte peut comporter un biais de perception. Cependant, la concordance entre les données quantitatives et les témoignages de terrain renforce la validité des interprétations. Malgré ces limites, cette étude constitue la première analyse empirique documentée de l'effet des sanctions économiques sur la qualité de la prise en charge nutritionnelle hospitalière au Niger et en Afrique de l'Ouest.

Les résultats révèlent une augmentation significative du nombre d'admissions d'enfants atteints de malnutrition aiguë sévère et une hausse de la mortalité hospitalière pendant le semestre post-sanctions de 2023. Ces changements coïncident avec la période de restriction commerciale et financière imposée au Niger après juillet 2023, suggérant un impact indirect mais tangible des sanctions sur la qualité des soins. Les ruptures prolongées d'intrants thérapeutiques (F75, F100, Plumpy'Nut, Resomal) et la stagnation des effectifs hospitaliers ont vraisemblablement contribué à cette dégradation. L'étude souligne un déséquilibre persistant entre l'offre et la demande de soins nutritionnels. Elle appelle à une redistribution plus équitable et planifiée des ressources humaines afin de réduire les inégalités interrégionales et de renforcer l'efficacité du dispositif national de prise en charge de la malnutrition aiguë sévère. Selon le modèle conceptuel de Donabedian, la qualité des soins dépend de trois dimensions interconnectées : structure, processus et résultats [7]. Dans ce cadre, la défaillance structurelle observée (manque de personnel, ruptures d'intrants) a perturbé les processus de soins (retards, rationnement, surcharge) et conduit à des résultats cliniques défavorables (hausse de la mortalité).

Les effets observés au Niger rejoignent ceux décrits dans d'autres contextes soumis à des sanctions économiques. En Iran, l’étude de Kokabisaghi [11] ont montré une réduction de l'accès aux médicaments essentiels après les sanctions de 2012, avec une augmentation des complications liées aux maladies chroniques. De même, Ghiasi *et al*. [13] ont décrit les pénuries d'insuline et d'antibiotiques comme des conséquences directes des restrictions bancaires. En Syrie, Human Rights Watch [14] et l'OMS (2021) [15] ont documenté les retards dans la livraison des intrants humanitaires et l'aggravation de la malnutrition infantile dans les zones hospitalières isolées. Au Venezuela, Rosales *et al*. [16] ont mis en évidence une hausse de la malnutrition aiguë chez les enfants liée à l'effondrement économique et à la réduction de l'aide internationale. Le cas nigérien présente des similitudes structurelles avec ces pays, mais avec un effet plus rapide et concentré dans le temps. En quelques mois, les sanctions ont engendré une désorganisation logistique majeure et une surcharge du personnel, révélant la faible résilience structurelle du système de santé national. Ces observations confirment les analyses de Kruk *et al*. [25], selon lesquelles les systèmes de santé fragiles des pays à faible revenu sont les plus vulnérables aux chocs économiques et politiques.

Malgré la crise, certains CRENI notamment à Zinder et Dosso ont maintenu des taux de guérison supérieurs à 90%, conformes aux standards SPHERE. Cette performance s'explique par la mobilisation rapide du ministère de la santé et des partenaires humanitaires (UNICEF, PAM, OMS), une stabilité relative du personnel et une meilleure coordination régionale. Ces résultats illustrent la capacité d'adaptation locale, en cohérence avec les travaux de Bhandari *et al*. [26] sur la résilience des hôpitaux africains en situation de crise. Ils confirment que la résilience du système de santé dépend autant de la gouvernance locale et du leadership clinique que des ressources matérielles.

Sur le plan programmatique, les résultats de cette étude appellent à plusieurs actions prioritaires: exempter les produits médicaux et nutritionnels essentiels de tout régime de sanction, conformément aux recommandations de l'OMS et du Haut-Commissariat aux droits de l'Homme [17]; renforcer la planification des ressources humaines et logistiques dans les services hospitaliers spécialisés ; mettre en place un système national d'alerte précoce pour anticiper les ruptures d'intrants et intégrer la surveillance de la qualité des soins nutritionnels dans les dispositifs de suivi sanitaire. Ces mesures rejoignent les conclusions de Garfield *et al*. [12] sur la nécessité de préserver les services vitaux face aux mesures coercitives internationales, ainsi que celles de De Maio *et al*. [27] sur la protection du droit à la santé en contexte de sanctions.

## Conclusion

Cette étude rétrospective multicentrique met en évidence un impact défavorable des sanctions économiques imposées au Niger en 2023 sur la qualité de la prise en charge hospitalière de la malnutrition aiguë sévère chez l'enfant. L'augmentation des admissions, associée à une baisse des taux de guérison et à une hausse de la mortalité hospitalière, traduit une fragilisation du dispositif de prise en charge nutritionnelle dans un contexte de crise économique et institutionnelle. Les ruptures d'intrants thérapeutiques essentiels et les contraintes sur les ressources humaines ont joué un rôle central dans cette dégradation. Les disparités régionales observées montrent toutefois une résilience variable des structures sanitaires, certaines ayant mieux maintenu leurs performances grâce à une organisation locale plus efficace et à l'appui des partenaires humanitaires. Conformément au modèle de Donabedian, ces résultats illustrent comment les perturbations structurelles induites par les sanctions affectent les processus de soins et se traduisent par des résultats cliniques défavorables. La protection des services de santé essentiels, notamment ceux dédiés à la prise en charge de la malnutrition aiguë sévère, doit constituer une priorité en période de sanctions économiques. Le renforcement de la résilience logistique, la sécurisation des approvisionnements et une meilleure planification des ressources humaines sont indispensables pour limiter l'impact de telles crises sur la survie infantile.

### Etat des connaissances sur le sujet

- Les sanctions économiques ont souvent des effets indirects sur la santé publique, en perturbant l'accès aux médicaments, aux intrants nutritionnels et au financement du système de santé;
- Les études menées en Iran, en Syrie et au Venezuela ont montré une dégradation de la qualité des soins et une augmentation de la morbidité et de la mortalité infantiles dans les périodes suivant les sanctions;
- En Afrique subsaharienne, il existe peu de données empiriques sur l'impact des sanctions économiques sur la prise en charge hospitalière de la malnutrition aiguë sévère.

### Contribution de notre étude à la connaissance

- Cette étude fournit la première analyse empirique documentée en Afrique de l'Ouest sur l'impact des sanctions économiques sur la qualité de la prise en charge nutritionnelle hospitalière;
- Elle met en évidence une corrélation entre les sanctions économiques, la rupture des intrants thérapeutiques et la hausse de la mortalité chez les enfants atteints de malnutrition aiguë sévère;
- Elle révèle des disparités régionales significatives dans la résilience des structures hospitalières, soulignant le rôle déterminant de la gouvernance locale et du soutien des partenaires dans la continuité des soins.

## Remerciements

Les auteurs expriment leur profonde gratitude aux médecins pédiatres et aux responsables des CRENI des régions de Dosso, Maradi, Niamey, Tahoua, Tillabéry et Zinder pour leur précieuse collaboration dans la collecte et la validation des données. Ils remercient également les agents de santé et les équipes de terrain qui ont contribué avec rigueur au remplissage des fiches d'extraction des données et à la consolidation des registres hospitaliers. Leur engagement et leur disponibilité ont été essentiels à la réussite de cette étude menée dans un contexte particulièrement difficile.
